# Prevalence and factors associated with underweight, overweight and obesity among women of reproductive age in India

**DOI:** 10.1186/s41256-019-0117-z

**Published:** 2019-09-06

**Authors:** Gulam Muhammed Al Kibria, Krystal Swasey, Md Zabir Hasan, Atia Sharmeen, Brendan Day

**Affiliations:** 1 Department of Epidemiology and Public Health, University of Maryland School of Medicine, 655 West Baltimore Street, Baltimore, MD-21201 USA; 2 Department of International Health, Johns Hopkins Bloomberg School of Public Health, Baltimore, MD-21205 USA; 3 School of Community Health and Policy, Morgan State University, Baltimore, MD-21251 USA

**Keywords:** Body weight, Underweight, Overweight, Obesity, India, Prevalence, Factor, Correlate, Body mass index, Prevalence, Women

## Abstract

**Introduction:**

Although the prevalence of underweight is declining among Indian women, the prevalence of overweight/obesity is increasing. This study examined the prevalence and factors associated with underweight and overweight/obesity among reproductive-aged (i.e., 15–49 years) women in India.

**Methods:**

This cross-sectional study analyzed data from the 2015–16 National Family Health Survey. The Asian and World Health Organization (WHO) recommended cutoffs for body mass index (BMI) were used to categorize body weight. The Asian and WHO BMI cutoffs for combined overweight/obesity were ≥ 23 and ≥ 25 kg/m^2^, respectively. Both recommendations had the same cutoff for underweight, < 18.5 kg/m^2^. After prevalence estimation, logistic regression was applied to investigate associated factors.

**Results:**

Among 647,168 women, the median age and BMI was 30 years and 21.0 kg/m^2^, respectively. Based on the Asian cutoffs, the overall prevalence of underweight was 22.9%, overweight was 22.6%, and obesity was 10.7%, compared to 15.5% overweight and 5.1% obesity as per WHO cutoffs. The prevalence and odds of underweight were higher among young, nulliparous, contraceptive non-user, never-married, Hindu, backward castes, less educated, less wealthy, and rural women. According to both cutoffs, women who were older, ever-pregnant, ever-married, Muslims, castes other than backwards, highly educated, wealthy, and living in urban regions had higher prevalence and odds of overweight/obesity.

**Conclusion:**

The prevalence of both non-normal weight categories (i.e., underweight and overweight/obesity) was high. A large proportion of women are possibly at higher risks of cardiovascular and reproductive adverse events due to these double nutrition burdens. Implementing large-scale interventions based on these results is essential to address these issues.

**Electronic supplementary material:**

The online version of this article (10.1186/s41256-019-0117-z) contains supplementary material, which is available to authorized users.

## Introduction

Overweight/obesity is a leading risk factor for global death and disability, and is associated with various non-communicable diseases including hypertension, diabetes, cancer, and cardiovascular disorders [1–3]. Globally, about one-third of adults are overweight/obese and about 10% of adults are underweight [4, 5]. Due to differences in biological (e.g., hormones) and behavioral characteristics (e.g., food deprivation during childhood and insufficient physical activity), females are more prone to being underweight, overweight and obese compared to their male counterparts [6–9]. Women with extreme body weight categories (i.e., underweight and overweight/obesity) suffer from infertility and adverse perinatal outcomes including abortion, preterm birth, and neonatal mortality [10–13]. Maternal obesity is associated with childhood obesity as well [14, 15]. Recent estimates suggest that the proportion of overweight/obese women is increasing alarmingly in most low- and middle-income countries (LMICs) due to current demographic transitions in these countries [5, 6]. For instance, a recent study conducted by Chowdhury et al. found that the prevalence of overweight/obesity increased from 9 to 39% in Bangladesh [16]. Another study by Vaidya et al. had similar results in Nepal [17].

With a population over 1 billion people, India is no exception to the trend of rising prevalence of overweight/obesity [18, 19]. This country is dealing with the double nutrition burden of underweight and overweight/obesity, and although among women of reproductive age, the prevalence of underweight has declined from 36% in 2005–06 to 23% in 2015–16, the prevalence of overweight/obesity has increased from 13% in 2005–06 to 21% in 2015–16 [19, 20]. In addition, more than half of the women in India are of reproductive age (i.e., 15–49 years), which represents about 250 million women [21]. To improve maternal and child health as well as the nutritional status of the overall population, it is particularly important to evaluate the nutritional status of reproductive-aged women. However, few studies have investigated prevalence and correlates of underweight and overweight/obesity among women in this age group with a nationally representative dataset in India. In this study, we address these existing gaps in the literature by investigating the prevalence and associated factors of extreme body weight categories among women of reproductive age in India.

## Methods

### Data source

This cross-sectional study used data from the 2015–16 National Family Health Survey (NFHS-4). The NFHS-4 was a nationally-representative survey and covered all states to obtain data on major health indicators in India, including maternal and child health indicators. The International Institute for Population Sciences (IIPS) implemented this survey from January 2015 to December 2016. In-person household interviews were conducted. The ethical approval for the survey was provided by Institutional Review Boards from the IIPS and ICF International. Verbal informed consent was obtained from respondents aged ≥18 years. If the respondent’s age was 15–17 years, consent was obtained from a legal guardian in addition to assent from the respondent. Details of this survey including methodologies, data collection, sample size, and findings are reported elsewhere [20]. The electronic approval to use the data was obtained from ICF International, Rockville, Maryland, USA in October 2018.

Briefly, the NFHS-4 involved two-stage sampling. The survey used the 2011 census as the sampling frame. Villages and census enumeration blocks (CEBs) served as the primary sampling units (PSUs) in rural and urban areas, respectively. With the probability proportional to size (PPS), villages were selected from the sampling frame. Based on the estimated number of households in a village, three substrata were created. Next, two more substrata were created based on the proportion of people representing scheduled castes and scheduled tribes (SCs/STs). The first three substrata were then crossed with the second two substrata to create six equal-sized strata. In urban regions, based on the proportion of SC/ST population, the CEBs were sorted. Then, the PPS sampling was used to select sample CEBs [20].

Complete mapping and listing of households were done in all PSUs. PSUs with ≥300 households were segmented into 100–150 households. Using systematic sampling with PPS segments, two segments were selected from those PSUs (i.e., PSUs with ≥300 households). Thus, either a PSU or a PSU segment made a cluster. In every selected cluster of both regions, 22 households were selected with systematic sampling. The total number of selected, occupied, and interviewed households was 628,900, 616,346, and 601,509, respectively. The overall response rate was 98% [20].

### Study variables

Body weight categories are commonly reported by body mass index (BMI). This is the ratio of weight (in kilograms), and height squared (in meters), usually expressed as kg/m^2^. Although the BMI cutoff to classify underweight is almost universal (i.e., < 18.5 kg/m^2^), two cutoffs are used to classify overweight and obesity [22]. The World Health Organization (WHO) uses the BMI cutoffs of 25–29.9 and ≥ 30 kg/m^2^ to categorize overweight and obesity, respectively. Since Asian people have higher cardiovascular and diabetes risks with a lower BMI, the suggested cutoffs for Asian people are 23–27.4 kg/m^2^ for overweight and ≥ 27.5 kg/m^2^ for obesity [22]. Considering the importance of both cutoffs, this study reported the prevalence and associated factors based on both cutoffs.

In this survey, the Seca 874 digital scale was used to measure weight and the Seca 213 stadiometer was used to measure height [20]. Trained survey staff obtained the measurements for a single time. BMI was rounded to the nearest hundredth decimal place. All pregnant women were excluded from prevalence estimates [20]. Explanatory variables were selected based on published reports and the dataset’s structure. Participants reported their age, sex, marital status, education level (i.e., no formal education, primary, secondary, and college or above), current hormonal contraceptive use, castes (i.e., SC, ST, other backward classes or others), and religion (i.e., Hindu, Muslim or others). The wealth status was obtained by principal component analysis of basic household construction materials and households elements [20]. Regarding location, place (i.e., rural or urban) and region of residence was obtained. Additional file 1: Table S1 describes all study variables.

### Data analysis

Stata 14.0 (Stata Corporation, College Station, Texas) was used to analyze data. Respondents’ background characteristics were reported by their weight classification according to both cutoffs. After assessing the normality of continuous variables, median and interquartile ranges (IQR) were used to describe them; categorical variables were reported by weighted numbers and percentages. The overall weighted prevalence (with 95% confidence intervals [CIs]) of underweight, overweight and obesity was reported based on background characteristics with both recommended cutoffs. Then, using ‘normal weight’ as the reference category of both cutoffs, simple and multivariable logistic regression analyses were conducted to investigate the associated factors of ‘underweight’ and ‘combined overweight/obesity’. Variables significant in unadjusted analysis were considered for incorporation into the multivariable analysis. Crude odds ratios (CORs) and adjusted odds ratios (AORs) were reported separately for both cutoffs. Multicollinearity was assessed by variance inflation factors (VIF); explanatory variables with VIF ≥10 were considered for removal from the multivariable model. We accounted for the cluster-sampling design of the NFHS-4 to obtain all weighted prevalence and associated factors.

## Results

Table 1 shows the background characteristics of the respondents. Among 647,168 women, 148,115, 215,652, and 133,748 were underweight, overweight/obese as per the Asian cutoff, and overweight/obese as per the WHO cutoff, respectively. The median age of the women was 30 years (IQR: 22–38), the underweight participants had a lower median age compared to overweight/obese women as per both cutoffs. About 70% of the women were pregnant at least once in their life. The overall proportion of contraceptive-using women was 4.5%. Overweight/obese women as per both Asian and WHO cutoffs had a higher proportion of contraceptive users compared to underweight women, 5.3, 5.1, and 3.1%, respectively. Approximately 23.8% of women were never-married; they composed a larger proportion of underweight women compared to overweight/obese women. The proportion of Hindu respondents was 80.7%; the underweight women had the highest proportion of Hindu women. Similarly, about 73.0% of respondents were from 1 of the 3 backward classes. Although the overweight/obese women as per both cutoffs had a higher proportion of women from upper wealth quintiles, the underweight women had a higher share from the lower two wealth quintiles. More than three-fourths of the underweight women were from rural areas (76.7%), while around half of the overweight/obese women were from rural areas (52.1 and 47.8% according to Asian and WHO cutoffs, respectively). About one-fourth of the women were from the Northern region (23.2%).

**Table 1:** Background characteristics of the survey participants according to body weight categories classified by guidelines^1^

Characteristics	Underweight (*n* = 148,115)	Asian Classification		WHO Classification		Overall (*n* = 647,168)
		Normal weight (*n* = 283,402)	Overweight/obese (*n* = 215,652)	Normal weight (*n* = 365,305)	Overweight/obese (*n* = 133,748)	
BMI, Median (IQR), kg/m^2^	17.3 (16.4–17.9)	20.6 (19.6–21.7)	25.6 (24.1–28.1)	21.2 (19.9–22.7)	27.5 (26.0–29.8)	21.0 (28.7–23.9)
Age (in years)						
Median (IQR)	24 (18–33)	28 (21–37)	35 (28–42)	29 (22–38)	36 (29–42)	30 (22–38)
15–19	47,783 (32.3)	55,595 (19.6)	10,623 (4.9)	61,395 (16.8)	4823 (3.6)	114,001 (17.6)
20–29	52,033 (35.1)	100,682 (35.5)	53,112 (24.6)	124,504 (34.1)	29,291 (21.9)	205,828 (31.8)
30–39	27,991 (18.9)	73,261 (25.9)	77,417 (35.9)	101,255 (27.7)	49,424 (37.0)	178,670 (27.6)
40–49	20,307 (13.7)	53,863 (19.0)	74,499 (34.5)	78,151 (21.4)	50,211 (37.5)	148,669 (23.0)
Parity						
Never pregnant	66,779 (45.1)	94,769 (33.4)	32,473 (15.1)	110,326 (30.2)	16,917 (12.6)	194,021 (30.0)
1–4	70,407 (47.5)	166,158 (58.6)	166,657 (77.3)	226,036 (61.9)	106,778 (79.8)	403,221 (62.3)
≥ 5	10,929 (7.4)	22,475 (7.9)	16,522 (7.7)	28,943 (7.9)	10,054 (7.5)	49,926 (7.7)
Hormonal contraceptive use						
No	143,494 (96.9)	270,436 (95.4)	204,148 (94.7)	347,672 (95.2)	126,913 (94.9)	618,079 (95.5)
Yes	4620 (3.1)	12,966 (4.6)	11,503 (5.3)	17,633 (4.8)	6836 (5.1)	29,089 (4.5)
Marital status						
Never married	57,503 (38.8)	75,329 (26.6)	20,970 (9.7)	86,160 (23.6)	10,139 (7.6)	153,803 (23.8)
Married	85,323 (57.6)	196,289 (69.3)	183,291 (85.0)	263,236 (72.1)	116,344 (87.0)	464,904 (71.8)
Widowed	3806 (2.6)	8541 (3.0)	8751 (4.1)	11,69 5 (3.2)	5597 (4.2)	21,098 (3.3)
Divorced/separated	1482 (1.0)	3242 (1.1)	2640 (1.2)	4214 (1.2)	1668 (1.2)	7364 (1.1)
Religion						
Hindu	123,208 (83.2)	230,707 (81.4)	168,637 (78.2)	296,040 (81.0)	103,303 (77.2)	522,551 (80.7)
Muslim	18,885 (12.8)	37,055 (13.1)	31,826 (14.8)	48,207 (13.2)	20,675 (15.5)	87,767 (13.6)
Others	6022 (4.1)	15,640 (5.5)	15,188 (7.0)	21,058 (5.8)	9771 (7.3)	36,851 (5.7)
Caste						
Scheduled caste	33,335 (22.5)	59,813 (21.1)	38,682 (17.9)	75,757 (20.7)	22,738 (17.0)	131,830 (20.4)
Scheduled tribe	18,807 (12.7)	29,040 (10.2)	11,446 (5.3)	34,574 (9.5)	5912 (4.4)	59,293 (9.2)
Other backward class	64,521 (43.6)	123,023 (43.4)	94,036 (43.6)	158,571 (43.4)	58,487 (43.7)	281,579 (43.5)
Other	31,452 (21.2)	71,526 (25.2)	71,488 (33.1)	96,403 (26.4)	46,611 (34.8)	174,466 (27.0)
Education level						
No formal education	44,172 (29.8)	83,099 (29.3)	52,078 (24.1)	104,983 (28.7)	30,195 (22.6)	179,349 (27.7)
Primary	17,831 (12.0)	35,173 (12.4)	28,165 (13.1)	45,712 (12.5)	17,627 (13.2)	81,169 (12.5)
Secondary	73,243 (49.4)	130,305 (46.0)	102,343 (47.5)	167,546 (45.9)	65,102 (48.7)	305,891 (47.3)
Higher	12,870 (8.7)	34,825 (12.3)	33,065 (15.3)	47,064 (12.9)	20,825 (15.6)	80,759 (12.5)
Wealth quintile						
Poorest	40,704 (27.5)	57,818 (20.4)	15,091 (7.0)	66,288 (18.1)	6621 (5.0)	113,613 (17.6)
Poorer	37,467 (25.3)	61,707 (21.8)	27,799 (12.9)	75,087 (20.6)	14,420 (10.8)	126,973 (19.6)
Middle	30,836 (20.8)	60,301 (21.3)	42,593 (19.8)	77,845 (21.3)	25,049 (18.7)	133,730 (20.7)
Richer	23,480 (15.9)	54,984 (19.4)	59,212 (27.5)	75,429 (20.6)	38,767 (29.0)	137,676 (21.3)
Richest	15,628 (10.6)	48,592 (17.1)	70,957 (32.9)	70,657 (19.3)	48,892 (36.6)	135,177 (20.9)
Place of residence						
Urban	34,549 (23.3)	84,952 (30.0)	103,301 (47.9)	118,404 (32.4)	69,849 (52.2)	222,802 (34.4)
Rural	113,565 (76.7)	198,450 (70.0)	112,350 (52.1)	246,901 (67.6)	63,899 (47.8)	424,366 (65.6)
Region						
Central	15,615 (10.5)	27,343 (9.6)	12,988 (6.0)	32,963 (9.0)	7368 (5.5)	55,945 (8.6)
Eastern	37,947 (25.6)	66,774 (23.6)	38,159 (17.7)	83,038 (22.7)	21,894 (16.4)	142,880 (22.1)
Northeastern	4985 (3.4)	11,400 (4.0)	6187 (2.9)	14,325 (3.9)	3262 (2.4)	22,572 (3.5)
Northern	32,224 (21.8)	67,841 (23.9)	50,114 (23.2)	87,417 (23.9)	30,538 (22.8)	150,179 (23.2)
Southern	25,181 (17.0)	57,178 (20.2)	65,886 (30.6)	79,213 (21.7)	43,851 (32.8)	148,245 (22.9)
Western	32,162 (21.7)	52,867 (18.7)	42,319 (19.6)	68,349 (18.7)	26,836 (20.1)	127,348 (19.7)

1. Numbers and column percentages unless otherwise specified

2. Asian and WHO classifications categorize combined overweight/obesity as BMI ≥23 and ≥ 25 Kg/m^2^, respectively. Both classifications categorize underweight as BMI <18.5 Kg/m^2^.

*BMI* Body mass index, *IQR* Inter-quartile range, *WHO* World Health Organization

**Fig. 1:**
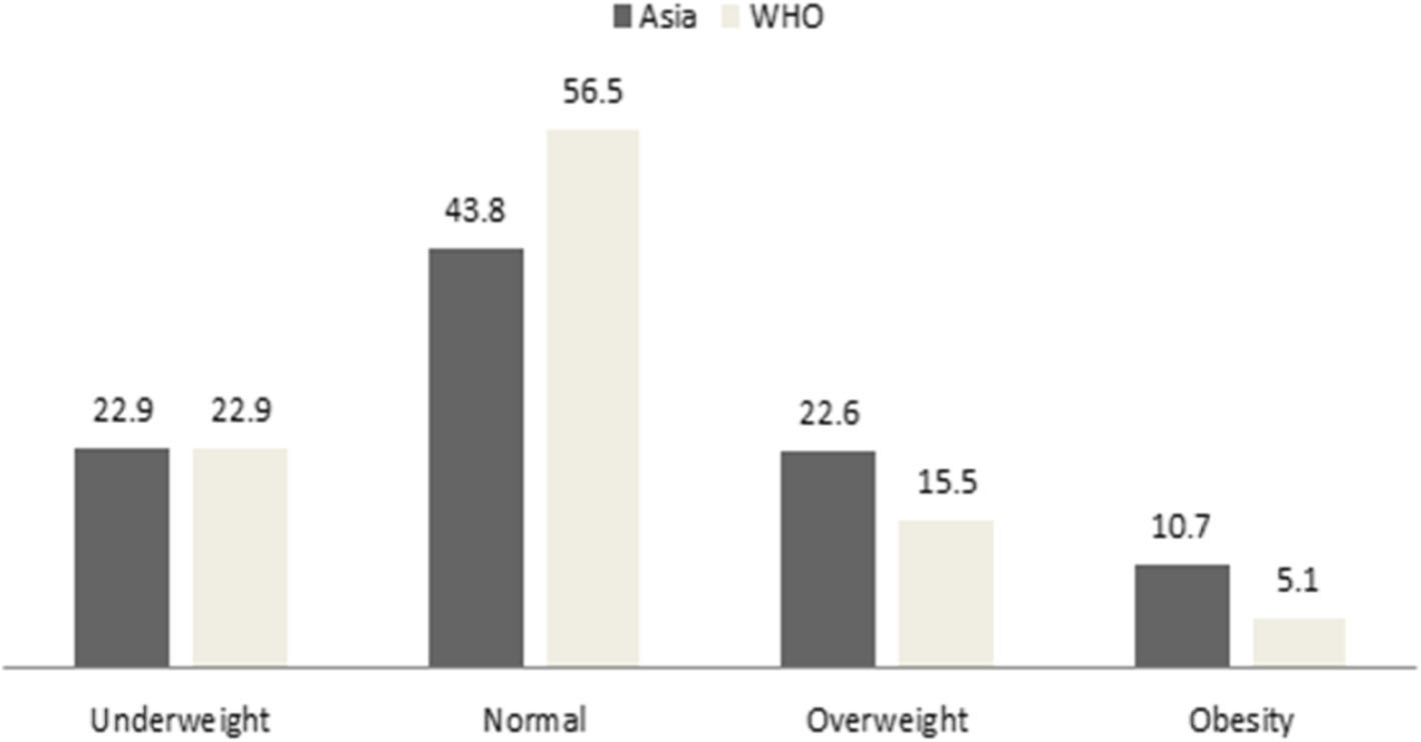
Prevalence (%) of body weight categories according to Asian and World Health Organization cutoffs”

Table 2 describes the prevalence according to different cutoffs. The prevalence of underweight, overweight and obesity as per the Asian cutoffs, and overweight and obesity as per the WHO cutoffs was 22.9% (95% CI: 22.7–23.1), 22.6% (95% CI: 22.5–22.8), 10.7% (95% CI: 10.5–10.8), 15.5% (95% CI: 15.4–15.7), and 5.1% (95% CI: 5.0–5.3), respectively. The prevalence of underweight declined with age while the prevalence of overweight/obesity increased with age as per both cutoffs. Ever-pregnant women had an increased prevalence of overweight/obesity compared to never-pregnant women as per both cutoffs. According to both the Asian and WHO cutoffs, women who reported that they were using a hormonal contraceptive had a higher prevalence of overweight and obesity while the prevalence of underweight was higher among women who were not using a hormonal contraceptive. The 3 backward classes (i.e., scheduled caste, scheduled tribe, and other backward classes) had increased prevalence of underweight, although classes other than these backward classes had increased prevalence of overweight/obesity as per both Asian and WHO cutoffs. As per both cutoffs, from the poorest to the richest wealth quintile, the prevalence of overweight and obesity increased; however, the prevalence of underweight was in reverse direction (i.e., decreased). Education level showed similar trends in prevalence. In urban regions, the Asian cutoffs’ prevalence was 28.6% (95% CI: 28.2–29.1) for overweight and 17.7% (95% CI: 17.3–18.1) for obesity, while the WHO cutoffs’ prevalence was 22.2% (95% CI: 21.8–22.6) for overweight and 9.1% (95% CI: 8.8–9.4) for obesity; the proportion of people with overweight/obesity was higher in urban regions compared to rural regions as per both cutoffs. The prevalence of underweight was higher in rural regions compared to urban regions (26.8% vs 15.5%). The highest prevalence of underweight was observed in Central region, 27.9% (95% CI: 27.5–28.4). Figure 1 and Additional file 1: Fig. S1 summarized the overall prevalence.

**Table 2:** Prevalence (with 95% CI) of underweight, overweight and obesity according to BMI categorization among women of reproductive age in India

Characteristics	Underweight^1^, %	Overweight, %		Obesity, %	
		Asian^2^	WHO^3^	Asian^2^	WHO^3^
Age (years)					
15–19	41.9 (41.5–42.4)	7.5 (7.2–7.7)	3.4 (3.2–3.6)	1.8 (1.7–2.0)	0.8 (0.7–0.9)
20–29	25.3 (25.0–25.6)	19.3 (19.0–19.6)	11.3 (11.1–11.6)	6.5 (6.3–6.7)	2.9 (2.8–3.0)
30–39	15.7 (15.4–15.9)	28.8 (28.4–29.1)	20.7 (20.4–21.0)	14.6 (14.2–14.9)	7.0 (6.8–7.2)
40–49	13.7 (13.4–13.9)	31.5 (31.2–31.9)	24.4 (24.1–24.8)	18.6 (18.2–18.9)	9.3 (9.1–9.6)
Parity					
Never pregnant	34.4 (34.1–34.8)	12.6 (12.4–12.8)	6.7 (6.6–6.9)	4.1 (4.0–4.3)	2.0 (1.9–2.1)
1–4	17.5 (17.3–17.7)	27.4 (27.2–27.7)	19.8 (19.5–20.0)	13.9 (13.7–14.1)	6.7 (6.6–6.9)
≥ 5	21.9 (21.4–22.4)	22.9 (22.5–23.4)	15.4 (14.9–15.8)	10.1 (9.8–10.5)	4.8 (4.5–5.0)
Hormonal contraceptive use					
No	23.2 (23.0–23.4)	22.4 (22.2–22.6)	15.4 (15.2–15.6)	10.6 (10.5–10.8)	5.1 (5.0–5.3)
Yes	15.9 (15.2–16.6)	28.0 (27.2–28.9)	18.3 (17.6–19.1)	11.5 (10.9–12.2)	5.2 (4.7–5.7)
Marital status					
Never married	37.4 (37.0–37.8)	10.7 (10.5–11.0)	5.2 (5.0–5.4)	2.9 (2.8–3.1)	1.4 (1.3–1.5)
Married	18.4 (18.2–18.5)	26.4 (26.1–26.6)	18.8 (18.5–19.0)	13.1 (12.9–13.3)	6.3 (6.1–6.4)
Widowed	18.0 (17.3–18.8)	27.2 (26.2–28.2)	19.2 (18.4–20.1)	14.3 (13.4–15.2)	7.3 (6.6–8.0)
Divorced/separated	20.1 (18.8–21.5)	24.2 (22.6–25.8)	17.3 (15.9–18.8)	11.7 (10.4–13.0)	5.3 (4.5–6.3)
Religion					
Hindu	23.6 (23.4–23.8)	22.2 (22.0–22.4)	15.0 (14.8–15.2)	10.0 (9.9–10.2)	4.8 (4.6–4.9)
Muslim	21.5 (21.0–22.0)	23.3 (22.8–23.8)	17.1 (16.6–17.5)	13.0 (12.5–13.5)	6.5 (6.2–6.8)
Others	16.3 (15.6–17.1)	27.1 (26.4–27.8)	19.2 (18.5–19.9)	14.2 (13.5–14.9)	7.3 (6.8–7.8)
Caste					
Scheduled caste	25.3 (24.9–25.7)	20.9 (20.5–21.3)	13.4 (13.1–13.8)	8.4 (8.1–8.8)	3.8 (3.6–4.0)
Scheduled tribe	31.7 (31.1–32.4)	15.0 (14.5–15.5)	8.1 (7.7–8.4)	4.3 (4.0–4.6)	1.9 (1.7–2.1)
Other backward class	22.9 (22.7–23.2)	22.7 (22.4–23.0)	15.6 (15.4–15.8)	10.7 (10.5–10.9)	5.2 (5.0–5.3)
Other	18.0 (17.7–18.4)	26.5 (26.1–26.9)	19.5 (19.1–19.8)	14.5 (14.2–14.9)	7.2 (7.0–7.5)
Education level					
No formal education	24.6 (24.3–24.9)	21.1 (20.8–21.4)	13.2 (12.9–13.4)	7.9 (7.7–8.2)	3.7 (3.5–3.8)
Primary	22.0 (21.5–22.4)	23.6 (23.1–24.0)	16.4 (16.0–16.9)	11.1 (10.7–11.5)	5.3 (5.0–5.6)
Secondary	23.9 (23.7–24.2)	22.1 (21.8–22.3)	15.7 (15.5–15.9)	11.4 (11.2–11.6)	5.6 (5.4–5.7)
Higher	15.9 (15.5–16.4)	27.4 (26.8–28.0)	19.2 (18.6–19.7)	13.5 (13.0–14.1)	6.6 (6.2–7.0)
Wealth quintile					
Poorest	35.8 (35.4–36.2)	11.2 (10.9–11.4)	5.0 (4.9–5.2)	2.1 (2.0–2.2)	0.8 (0.7–0.9)
Poorer	29.5 (29.1–29.9)	17.2 (16.9–17.5)	9.5 (9.3–9.8)	4.7 (4.5–4.9)	1.8 (1.7–2.0)
Middle	23.1 (22.7–23.4)	23.2 (22.8–23.5)	14.9 (14.6–15.2)	8.7 (8.4–8.9)	3.8 (3.6–4.0)
Richer	17.1 (16.7–17.4)	27.9 (27.5–28.3)	20.8 (20.4–21.2)	15.1 (14.8–15.5)	7.4 (7.1–7.6)
Richest	11.6 (11.2–12.0)	31.6 (31.1–32.1)	25.2 (24.8–25.7)	20.9 (20.4–21.4)	11.0 (10.6–11.3)
Place of residence					
Urban	15.5 (15.1–15.9)	28.6 (28.2–29.1)	22.2 (21.8–22.6)	17.7 (17.3–18.1)	9.1 (8.8–9.4)
Rural	26.8 (26.6–27.0)	19.5 (19.3–19.7)	12.0 (11.9–12.2)	7.0 (6.9–7.1)	3.1 (3.0–3.1)
Region					
Central	27.9 (27.5–28.4)	16.9 (16.5–17.2)	10.3 (10.0–10.6)	6.3 (6.1–6.6)	2.9 (2.8–3.1)
Eastern	26.5 (26.1–27.0)	19.6 (19.2–20.0)	12.3 (12.0–12.7)	7.1 (6.8–7.4)	3.0 (2.8–3.2)
Northeastern	22.1 (21.4–22.7)	21.5 (20.9–22.0)	12.2 (11.7–12.6)	5.9 (5.6–6.2)	2.3 (2.1–2.5)
Northern	21.4 (21.1–21.8)	22.8 (22.6–23.1)	15.2 (15.0–15.5)	10.5 (10.3–10.7)	5.1 (5.0–5.3)
Southern	17.0 (16.6–17.4)	28.4 (27.9–28.8)	21.5 (21.0–21.9)	16.1 (15.6–16.5)	8.1 (7.8–8.4)
Western	25.2 (24.7–25.8)	21.9 (21.4–22.3)	15.5 (15.0–15.9)	11.3 (10.9–11.8)	5.6 (5.3–5.9)
**Overall**	22.9 (22.7–23.1)	22.6 (22.5–22.8)	15.5 (15.4–15.7)	10.7 (10.5–10.8)	5.1 (5.0–5.3)

1. Both Asian and World Health Organization classifications categorize underweight as BMI <18.5 Kg/m^2^

2. Asian classification categorizes overweight and obesity as BMI ≥23 and ≥ 27.5 Kg/m^2^, respectively

3. World Health Organization classification categorizes overweight and obesity as BMI ≥25 and ≥ 30 Kg/m^2^, respectively

*CI* Confidence interval, *WHO* World Health Organization

Table 3 presents the CORs and AORs of the factors associated with underweight as per both cutoffs. With decreasing age, the odds of underweight increased, with the highest odds of underweight among the women of 15–19 years according to both Asian (AOR: 2.07, 95% CI: 2.00–2.13) and WHO (AOR: 2.58, 95% CI: 2.51–2.66) cutoffs. The number of pregnancies also had a significant association with underweight. Women who were not using hormonal contraceptives had greater odds of underweight according to both Asian (AOR: 1.17, 95% CI: 1.13–1.21) and WHO (AOR: 1.20, 95% CI: 1.16–1.24) cutoffs. Although being a married woman was protective against underweight as per the Asian cutoff, being a never-married woman was a factor associated with increased underweight per both cutoffs. Both Muslim and Hindu women were more likely to be underweight compared to women belonging to other religions. Based on both cutoffs, all socioeconomic variables were significantly associated with underweight; women with lower household wealth quintiles, education level, and backward classes had positive association with underweight compared to women with the richest wealth quintile, higher education level and other classes, respectively. Rural women had increased odds of underweight as per both Asian (AOR: 1.06, 95% CI: 1.04–1.08) and WHO (AOR: 1.09, 95% CI: 1.07–1.11) cutoffs compared to urban women. Region of residence was also a significant variable.

**Table 3:** Determinants of underweight among women of reproductive age in India^1,2^

Characteristics	Asian				WHO			
	COR	(95% CI)	AOR	(95% CI)	COR	(95% CI)	AOR	(95% CI)
Age (in years)								
15–19	2.28 (2.22–2.34)		2.07 (2.00–2.13)		3.00 (2.91–3.08)		2.58 (2.51–2.66)	
20–29	1.37 (1.33–1.41)		1.53 (1.50–1.57)		1.61 (1.57–1.65)		1.81 (1.77–1.85)	
30–39	1.01 (0.98–1.04)		1.08 (1.06–1.11)		1.06 (1.03–1.09)		1.15 (1.12–1.17)	
40–49 (Ref.)	**1.0**		**1.00**		**1.00**		**1.00**	
Parity								
Never pregnant	1.45 (1.40–1.49)		0.82 (0.79–0.85)		1.60 (1.56–1.65)		0.80 (0.77–0.83)	
1–4	0.87 (0.85–0.90)		0.88 (0.86–0.91)		0.82 (0.80–0.85)		0.85 (0.83–0.88)	
≥ 5 (Ref.)	**1.00**		**1.00**		**1.00**		**1.00**	
Hormonal contraceptive use								
No	1.49 (1.41–1.57)		1.17 (1.13–1.21)		1.58 (1.49–1.66)		1.20 (1.16–1.24)	
Yes (Ref.)	**1.00**		**1.00**		**1.00**		**1.00**	
Marital status								
Never married	1.67 (1.52–1.83)		1.24 (1.16–1.33)		1.90 (1.74–2.07)		1.26 (1.18–1.35)	
Married	0.95 (0.87–1.04)		0.87 (0.82–0.93)		0.92 (0.84–1.01)		0.84 (0.79–0.90)	
Widowed	0.97 (0.88–1.08)		0.96 (0.90–1.04)		0.93 (0.84–1.02)		0.96 (0.89–1.03)	
Divorced/separated (Ref.)	**1.00**		**1.00**		**1.00**		**1.00**	
Religion								
Hindu	1.39 (1.31–1.46)		1.56 (1.51–1.60)		1.46 (1.38–1.54)		1.61 (1.56–1.65)	
Muslim	1.32 (1.25–1.41)		1.51 (1.47–1.57)		1.37 (1.29–1.45)		1.51 (1.47–1.56)	
Others (Ref.)	**1.00**		**1.00**		**1.00**		**1.00**	
Caste								
Scheduled caste	1.27 (1.23–1.31)		1.12 (1.10–1.15)		1.35 (1.31–1.39)		1.14 (1.12–1.16)	
Scheduled tribe	1.47 (1.42–1.53)		0.96 (0.94–0.99)		1.67 (1.61–1.73)		0.99 (0.97–1.01)	
Other backward class	1.19 (1.16–1.23)		1.08 (1.06–1.10)		1.25 (1.21–1.28)		1.10 (1.08–1.12)	
Others (Ref.)	**1.00**		**1.00**		**1.00**		**1.00**	
Education								
No formal education	1.44 (1.38–1.49)		1.35 (1.31–1.39)		1.54 (1.48–1.60)		1.41 (1.37–1.45)	
Primary	1.37 (1.31–1.43)		1.24 (1.20–1.28)		1.43 (1.37–1.49)		1.26 (1.22–1.30)	
Secondary	1.52 (1.47–1.58)		1.17 (1.14–1.20)		1.60 (1.54–1.66)		1.17 (1.14–1.20)	
Higher (Ref.)	**1.00**		**1.00**		**1.00**		**1.00**	
Wealth quintile								
Poorest	2.19 (2.09–2.29)		1.99 (1.93–2.05)		2.78 (2.65–2.91)		2.33 (2.26–2.40)	
Poorer	1.89 (1.80–1.98)		1.69 (1.64–1.73)		2.26 (2.15–2.36)		1.90 (1.85–1.95)	
Middle	1.59 (1.51–1.67)		1.42 (1.38–1.45)		1.79 (1.71–1.88)		1.53 (1.49–1.57)	
Richer	1.33 (1.26–1.39)		1.24 (1.20–1.27)		1.41 (1.34–1.48)		1.28 (1.25–1.32)	
Richest (Ref.)	1.00		1.00		1.00		1.00	
Place of residence								
Urban (Ref.)	**1.00**		**1.00**		**1.00**		**1.00**	
Rural	1.41 (1.37–1.45)		1.06 (1.04–1.08)		1.58 (1.53–1.62)		1.09 (1.07–1.11)	
Region								
Central	1.20 (1.17–1.24)		1.26 (1.23–1.29)		1.29 (1.25–1.32)		1.31 (1.28–1.33)	
Eastern	1.20 (1.16–1.23)		1.21 (1.19–1.23)		1.24 (1.20–1.28)		1.23 (1.20–1.25)	
Northeastern	0.92 (0.88–0.96)		0.81 (0.79–0.83)		0.94 (0.91–0.98)		0.80 (0.78–0.83)	
Northern (Ref.)	1.00		1.00		1.00		1.00	
Southern	0.93 (0.90–0.96)		1.15 (1.12–1.18)		0.86 (0.83–0.89)		1.12 (1.09–1.14)	
Western	1.28 (1.24–1.33)		1.55 (1.52–1.58)		1.28 (1.23–1.32)		1.59 (1.56–1.62)	

1. Asian and WHO classifications categorize normal weight as BMI 18.5–22.9 and 18.5–24.9 Kg/m^2^, respectively. Both classifications define underweight as the BMI of < 18.5 Kg/m^2^

2. The *p* values were below < 0.05 when the 95% confidence interval did not include 1

*AOR* Adjusted odds ratio, *COR* Crude odds ratio, *CI* Confidence interval, *WHO* World Health Organization

In Table 4, the results of logistic regression analyses to investigate potential correlates of overweight/obesity are presented. All variables that were associated with underweight were also associated with overweight/obesity as per both cutoffs. Women with the highest age (i.e., 40–49 years) had the highest odds of overweight/obesity as per both Asian (AOR: 5.00, 95% CI: 4.84–5.17) and WHO (AOR: 5.38, 95% CI: 5.15–5.61) cutoffs. Women with 1–4 parity had increased odds of overweight/obesity based on the Asian cutoff (AOR 1.11, 95% CI: 1.08–1.14), and both the 1–4 (AOR 1.13, 95% CI: 1.09–1.16) and ≥ 5 parity (AOR: 1.11, 95% CI: 1.07–1.16) had positive association with this outcome based on the WHO cutoff. Although women who were using hormonal contraceptives during the survey period had positive association with overweight/obesity as per the Asian cutoff (AOR: 1.05, 95% CI: 1.02–1.08), it had insignificant association as per the WHO cutoff (AOR: 0.99, 95% CI: 0.96–1.02). Marital status, religion, castes, education level, wealth status, place and region of residence also had significant relationships with overweight/obesity.

**Table 4:** Determinants of overweight/obesity among women of reproductive age in India^1,2^

Characteristics	Asian		WHO	
	COR (95% CI)	AOR (95% CI)	COR (95% CI)	AOR (95% CI)
Age (in years)				
15–19 (Ref.)	**1.00**	**1.00**	**1.00**	**1.00**
20–29	2.76 (2.66–2.87)	1.84 (1.79–1.90)	2.99 (2.84–3.16)	1.95 (1.87–2.03)
30–39	5.53 (5.33–5.73)	3.68 (3.56–3.79)	6.21 (5.89–6.55)	3.96 (3.80–4.13)
40–49	7.24 (6.97–7.51)	5.00 (4.84–5.17)	8.18 (7.75–8.63)	5.38 (5.15–5.61)
Parity				
Never pregnant (Ref.)	**1.00**	**1.00**	**1.00**	**1.00**
1–4	2.93 (2.86–3.00)	1.11 (1.08–1.14)	3.08 (2.99–3.18)	1.13 (1.09–1.16)
≥ 5	2.15 (2.07–2.22)	1.03 (0.99–1.07)	2.27 (2.17–2.36)	1.11 (1.07–1.16)
Hormonal contraceptive use				
No (Ref.)	**1.00**	**1.00**	**1.00**	**1.00**
Yes	1.18 (1.12–1.23)	1.05 (1.02–1.08)	1.06 (1.01–1.12)	0.99 (0.96–1.02)
Marital status				
Never married (Ref.)	**1.00**	**1.00**	**1.00**	**1.00**
Married	3.35 (3.27–3.45)	1.54 (1.49–1.59)	3.76 (3.62–3.90)	1.65 (1.59–1.72)
Widowed	3.68 (3.48–3.89)	1.38 (1.32–1.44)	4.07 (3.80–4.35)	1.50 (1.42–1.58)
Divorced/separated	2.92 (2.69–3.18)	1.29 (1.21–1.37)	3.36 (3.04–3.72)	1.49 (1.39–1.60)
Religion				
Hindu	**1.00**	**1.00**	**1.00**	**1.00**
Muslim	1.18 (1.14–1.21)	1.30 (1.28–1.33)	1.23 (1.18–1.28)	1.36 (1.34–1.39)
Others (Ref.)	1.33 (1.28–1.38)	1.18 (1.15–1.21)	1.33 (1.27–1.39)	1.13 (1.10–1.16)
Caste				
Scheduled caste	**1.00**	**1.00**	**1.00**	**1.00**
Scheduled tribe	0.61 (0.58–0.64)	0.84 (0.82–0.86)	0.57 (0.54–0.60)	0.78 (0.75–0.80)
Other backward class	1.18 (1.15–1.22)	0.99 (0.98–1.01)	1.23 (1.18–1.27)	1.00 (0.98–1.02)
Other (Ref.)	1.55 (1.50–1.60)	1.14 (1.12–1.16)	1.61 (1.55–1.67)	1.16 (1.13–1.19)
Education				
No formal education (Ref.)	**1.00**	**1.00**	**1.00**	**1.00**
Primary	1.28 (1.24–1.32)	1.18 (1.15–1.20)	1.34 (1.30–1.39)	1.19 (1.16–1.22)
Secondary	1.25 (1.22–1.28)	1.28 (1.26–1.30)	1.35 (1.32–1.39)	1.29 (1.26–1.32)
Higher	1.52 (1.46–1.57)	1.23 (1.20–1.27)	1.54 (1.48–1.60)	1.20 (1.17–1.24)
Wealth quintile				
Poorest (Ref.)	**1.00**	**1.00**	**1.00**	**1.00**
Poorer	1.73 (1.67–1.78)	1.53 (1.49–1.57)	1.92 (1.84–2.01)	1.69 (1.64–1.75)
Middle	2.71 (2.62–2.79)	2.19 (2.14–2.25)	3.22 (3.09–3.36)	2.60 (2.52–2.68)
Richer	4.13 (4.00–4.26)	3.03 (2.95–3.10)	5.15 (4.94–5.36)	3.74 (3.62–3.87)
Richest	5.59 (5.41–5.79)	3.90 (3.79–4.01)	6.93 (6.64–7.23)	4.83 (4.66–5.00)
Place of residence				
Urban	2.15 (2.10–2.20)	1.28 (1.26–1.30)	2.28 (2.22–2.34)	1.32 (1.30–1.34)
Rural (Ref.)	**1.00**	**1.00**	**1.00**	**1.00**
Region				
Central	0.64 (0.62–0.66)	0.75 (0.74–0.77)	0.64 (0.62–0.66)	0.76 (0.74–0.79)
Eastern	0.77 (0.75–0.80)	0.97 (0.95–0.99)	0.75 (0.73–0.78)	1.02 (0.99–1.04)
Northeastern	0.73 (0.71–0.76)	0.98 (0.96–1.00)	0.65 (0.62–0.68)	0.92 (0.89–0.94)
Northern (Ref.)	**1.00**	**1.00**	**1.00**	**1.00**
Southern	1.56 (1.51–1.61)	1.26 (1.23–1.28)	1.58 (1.53–1.64)	1.29 (1.27–1.32)
Western	1.08 (1.05–1.12)	0.85 (0.83–0.87)	1.12 (1.08–1.17)	0.87 (0.85–0.89)

1. Asian and WHO classifications categorize overweight/obesity as BMI ≥23 and ≥ 25 Kg/m^2^, respectively

2. The p values were below < 0.05 when the 95% confidence interval did not include 1

*AOR* Adjusted odds ratio, *COR* Crude odds ratio, *CI* Confidence interval, *WHO* World Health Organization

: *p* < 0.05,: *p* < 0.01,: *p* < 0.001

## Discussion

Using a large nationally representative sample, this study shows that although underweight remains a significant public health issue (affecting roughly 1 in 5 women), overweight/obesity now affects a similar or greater proportion of women depending on which cutoffs are used (1 in 5 women according to WHO cutoffs vs 1 in 3 women according to Asian cutoffs). Although the Asian cutoffs identified a greater proportion of women as overweight/obese, the associated factors were similar. We observed increased prevalence and odds of underweight among younger, never-pregnant, non-users of hormonal contraceptive, unmarried, backward classes, less educated, and less wealthy women. Most factors that had positive association with the prevalence and odds of underweight, had inverse (i.e., negative, protective against, or were in opposite direction) association with overweight/obesity.

The positive association between age and body weight could be due to the fact that increasing age is a known associated factor of overweight as well as for other non-communicable diseases [23]. Furthermore, advancing age is correlated with number of parity, another associated factor for overweight/obesity [24]. Women usually gain weight during pregnancy, which could be sustained for a lifetime if weight loss does not occur in the post-partum period [13, 25]. Additionally, never-married women had higher odds of underweight, and ever-married women had higher odds of overweight/obesity as per both cutoffs. The greater odds among ever-married women might be due to gestational weight gain but could also be influenced by increasing socioeconomic status and related factors. Similar to earlier studies, women who reported that they were using hormonal contraceptives during the survey period had increased prevalence of overweight/obesity compared to women who were not using hormonal contraceptives [26, 27]. In addition to the weight gain associated with hormonal contraceptive use, women who use hormonal contraceptives are more likely to be older, have children, or be married [28, 29]. These factors might have synergistic effects on the body weights of hormonal contraceptive-using women.

Socioeconomic variables such as urban residence, higher education level, and wealth status had positive association with overweight/obesity per both cutoffs. In contrast, rural women were more likely to be underweight. Women with higher education level are more likely to have higher wealth status than less-educated women [30]. Previous research from India and other South Asian countries have observed similar relationships [16, 31, 32]. People with a higher SES in developing countries usually follow more sedentary lifestyles or less labor-intensive occupations, and consume more energy due to their greater purchasing ability [33, 34]. These characteristics could result in increased body weight among these individuals. The increased prevalence of underweight among women with lower SES could result from consuming fewer calories and less nutritious foods. People with a lower SES might not be able to afford adequate food for themselves and their families and may lack knowledge regarding nutritious foods [34]. Differences in socioeconomic, dietary, and lifestyle factors could contribute to the differences in weight categories between castes and religions. For instance, a large proportion of Hindu people in India are vegetarians, and they consume less calorigenic foods compared to non-vegetarians [35, 36].

Our findings have considerable public health implications for a populated country like India, where more than one-sixth of the total world population lives and about half of the women are within their reproductive age [21]. Furthermore, considering the population size, this sample represents more than one-twelfth of the total women in the world. The combined prevalence of underweight, overweight and obesity was 56.2% as per the Asian cutoffs; in contrast, the WHO cutoffs found the combined prevalence as 43.5%. Lowering the cutoff reclassified a significant proportion of women as overweight/obese. However, due to higher health risks for Asian people at a lower BMI cutoff, these findings indicate that more than half of these women could be at an elevated risk of cardiovascular and reproductive health-related adverse consequences [22]. Moreover, programs targeting reduction of neonatal or childhood mortality may not be successful without addressing maternal nutrition issues, as maternal health is closely related to child health [10–13]. Although the prevalence of overweight/obesity categorized by the WHO-recommended cutoffs was lower than in high income countries, the prevalence of underweight was substantially higher than in wealthier countries [5, 6]. Among women who are at a greater risk of complications resulting from extreme BMIs, it is important to increase awareness to maintain a healthy weight; understanding the factors that are associated with higher prevalence or likelihood of both conditions are important in this context. All of these identified factors are also known correlates of body weights that have been established by a large number of earlier studies conducted in many LMICs including India [16, 18, 19, 31–33]. Our study reconfirmed the significance of these factors.

This study has several limitations. Since this dataset was cross-sectional, some observed factors might not be causally associated due to lack of evidence about temporal relationship. Some known associated factors including physical activity levels, dietary habits, nutritional factors, or other comorbid conditions were not adjusted due to limitations of the dataset. However, this study has several notable strengths. First, highly trained teams used standardized and validated instruments to obtain all measurements in NFHS-4. The survey had a large sample size and a high response rate. It covered rural and urban regions of all states. These findings may be generalizable to all women of reproductive age in India. To our knowledge, this is the first population-based study that reported prevalence and correlates of underweight and overweight/obesity among women of reproductive age in India as per two recommended cutoffs.

## Conclusion

Our results show that a large proportion of reproductive-aged women belong to non-normal BMI categories in India, placing them at increased risks of complications resulting from underweight or overweight/obesity. As the associated factors are similar regardless of cutoffs, addressing factors associated with a higher prevalence of these ‘non-normal’ BMI categories is crucial not only to combating the overall noncommunicable disease burden, but also for improving maternal and child health conditions.

## Additional file

Additional file 1: **Table S1.** Description of study variables. **Fig. S1**. Prevalence of different body mass index categories. (DOCX 171 kb)

## Data Availability

Data is available upon request from the ICF International website (https://dhsprogram.com/data/available-datasets.cfm). Dr. Kibria has full access to the data and takes responsibility for the accuracy of the data analysis.

## Abbreviations

AOR: Adjusted odds ratio
BMI: Body mass index
CEBs: Census enumeration blocks
CI: Confidence interval
COR: Crude odds ratio
EA: Enumeration area
NFHS: National Family and Health Survey
OR: Odds ratio
PSU: Primary sampling unit
SC: Scheduled castes
SES: Socioeconomic status
ST: Scheduled tribes
WHO: World Health Organization
