# Supplementary material for: Prevalence and factors associated with underweight, overweight and obesity among women of reproductive age in India
Source: Glob Health Res Policy. 2019 Sep 6;4:24. doi: 10.1186/s41256-019-0117-z (PMC6729094; doi:10.1186/s41256-019-0117-z)
Supplement: Supplementary file 1 — Table S1. Description of study variables. Fig. S1. Prevalence of different body mass index categories. (DOCX 171 kb) [file 41256_2019_117_MOESM1_ESM.docx]

| Additional file Table 1: Description of study variables | |
| --- | --- |
| Variable/characteristics | Description |
| Body mass index | Continuous variable; obtained by dividing weight (in Kilograms) by squares of height (in m^2^). |
| Underweight | Body mass index below 18.5 Kg/m^2^ |
| Overweight | Body mass index as 23-27.5 and 25-30 Kg/m^2^ as per the Asian and World Health Organization classifications, respectively. |
| Obesity | Body mass index as ≥27.5 and ≥30 Kg/m^2^ as per the Asian and World Health Organization classifications, respectively. |
| Age (in years) | Age of the respondents in years, 4 categories: 15-19, 20-29,30-39, and 40-49. |
| Parity | Number of pregnancy in the lifetime of a woman, 3 categories: never-pregnant (0), 1-4 and ≥5. |
| Contraceptive use | If a woman was using contraceptive at the period of survey; binary: no and yes. |
| Marital status | Marital status of a woman during the survey period: never-married, currently married, widowed and divorced/separated. |
| Religion | Religion of a woman during survey period; 3 categories: Hindu, Muslim and others. |
| Caste | Caste of a woman during survey period; 4 categories: Scheduled castes, scheduled tribes, other backward classes and others (i.e., classes other than backwards). |
| Education level | Education level of a woman during survey period; no formal education, primary (i.e., up to five completed years), secondary (i.e., up to ten completed years) and college or above (i.e., ≥12 years of education). |
| Wealth quintile | Household wealth status of a woman during survey period. Principal component analysis was used to obtain wealth status. The wealth status was stratified into quintiles: poorest, poorer, middle, richer and richest. |
| Place of residence | Residence of a woman during survey period; binary: rural or urban. |
| Region of residence | Administrative region where participants stayed during survey period: Northern (Chandigarh, Delhi, Haryana, Himachal Pradesh, Jammu and Kashmir, Punjab, and Rajasthan); Northeastern (Assam, Arunachal Pradesh, Manipur, Meghalaya, Mizoram, Nagaland, Tripura, and Sikkim); Central (Chhattisgarh, Madhya Pradesh, Uttarakhand, and Uttar Pradesh); Eastern (Bihar, Jharkhand, Odisha, and West Bengal); Western (Dadra and Nagar Haveli, Daman and Diu, Goa, Gujarat, and Maharashtra); and Southern (Andhra Pradesh, Karnataka, Kerala, Puducherry, Tamil Nadu, Telangana, Andaman and Nicobar Islands, and Lakshadweep). |


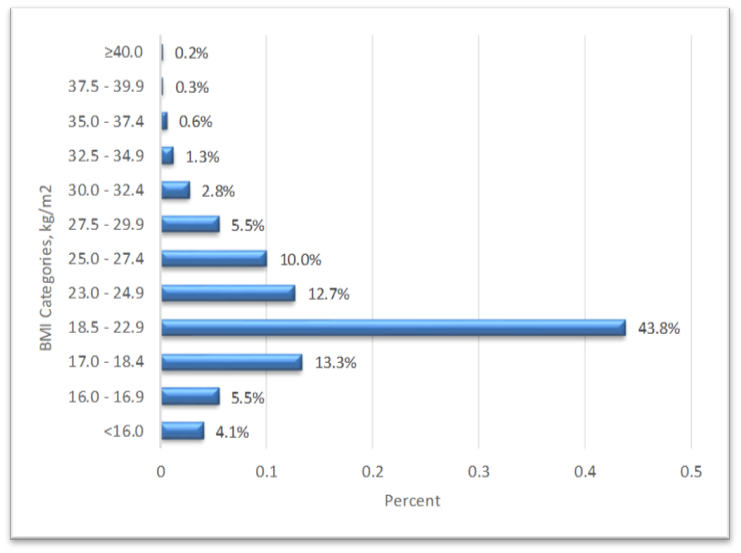


Additional file Figure 1: Prevalence of different body mass index categories
